# The impact of environmental enrichment on the murine inflammatory immune response

**DOI:** 10.1172/jci.insight.90723

**Published:** 2017-04-06

**Authors:** Samuel Brod, Thomas Gobbetti, Beatrice Gittens, Masahiro Ono, Mauro Perretti, Fulvio D’Acquisto

**Affiliations:** 1 William Harvey Research Institute, Barts and the London School of Medicine and Dentistry, Queen Mary University of London, London, England, United Kingdom.; 2 University of London Imperial College Science Technology & Medicine, Department of Life Science, Faculty of Natural Science, London, England, United Kingdom.

## Abstract

Living in a mentally and physically stimulating environment has been suggested to have a beneficial effect on the immune response. This study investigates these effects, utilizing a 2-week program of environmental enrichment (EE) and 2 models of acute inflammation: zymosan-induced peritonitis (ZIP) and the cecal ligation and puncture (CLP) model of sepsis. Our results revealed that following exposure to EE, mice possessed a significantly higher circulating neutrophil to lymphocyte ratio compared with control animals. When subject to ZIP, EE animals exhibit enhanced neutrophil and macrophage influx into their peritoneal cavity. Corresponding results were found in CLP, where we observed an improved capacity for enriched animals to clear systemic microbial infection. Ex vivo investigation of leukocyte activity also revealed that macrophages from EE mice presented an enhanced phagocytic capacity. Supporting these findings, microarray analysis of EE animals revealed the increased expression of immunomodulatory genes associated with a heightened and immunoprotective status. Taken together, these results provide potentially novel mechanisms by which EE influences the development and dynamics of the immune response.

## Abstract 2

A housing period as little as 2 weeks in an enriched environment with an increase in spatial and sensorial stimuli improved the ability of mice to clear infections.

## Introduction

Inflammation plays a pivotal role in the normal function of many living organisms, serving both as an initiator of the immune response and a key modulator of tissue homeostasis (1). Activated by a diversity of internal and external stimuli, inflammation aids in the clearance of pathogens, dead tissue, and foreign bodies as well as remodeling tissues to facilitate regrowth and repair.

Inflammation is distinct from other homeostatic mechanisms in that damage to self is an unavoidable and often necessary part of its process. As such, uncontrolled inflammation can be highly detrimental, leading to irreparable tissue damage and, potentially, autoimmune disease. While a long-studied field of research (2), recent innovations in cellular and molecular biology have greatly expanded our knowledge of the factors regulating (and dysregulating) the inflammatory response.

Such knowledge has allowed us to divide this process into several subtypes, such as silent inflammation (due to aging or lifestyle), sterile inflammation (e.g., gouty arthritis) (3), para-inflammation (e.g., macular degeneration) (4), and cellular stress responses (5), each with its own distinct effects and etiologies. Accordingly, we now appreciate that inflammation is a multifaceted and adaptive process, sensitive to alterations both internal and external to the host.

A growing number of studies have identified that nonheritable or environmental factors play a significantly greater role in influencing the inflammatory response than previously recognized (6). Multiple inflammatory and autoimmune diseases have been linked to circumstances such as diet (7), psychological state (8), living conditions (9), and socioeconomic status (10). While the growing availability of clinical and epidemiological data provides us with a broad picture of the incidence and outcomes of this cross-talk (11, 12), there is a strong need for further research into the mechanisms by which these factors achieve this influence.

Corresponding animal research in the field of neurology has utilized environmental enrichment (EE) — an experimental paradigm that attempts to model an active social, mental, and physical life in captive animals (13). Such research has revealed how a common feature of EE is its ability to bring about an improved capacity to return to a normal homeostatic state. For example, exposure to EE has been shown to mitigate or facilitate repair of neurological damage caused by chemical exposure (14), physical trauma (15), and neurodegenerative disease (16).

Alongside these neurological effects, a small number of investigations have reported the modulating effects EE has upon the immune response. For example, one study demonstrated that following 4 months in an enriched environment, mice show an improved response to infection with influenza type A, clearing the virus more quickly than animals housed in a standard environment (SE) (17). Further studies on mice have also suggested that EE may have specific immunomodulatory effects, enhancing leukocyte effector function (13) while decreasing levels of circulating inflammatory cytokines (18, 19). Such an unusual immune profile would appear likely to promote an equally unique inflammatory response. However, as of yet no direct research has investigated the effects of EE on classical experimental models of inflammatory disease.

We applied an established enrichment paradigm alongside classic experimental models of disease, exposing groups of male outbred CD1 mice to a 14-day EE regime and then assessing the scale and intensity of their immune response in the context of experimental sepsis or peritonitis. Our results revealed that EE has an enhancing effect on the murine immune inflammatory response. We found that housing in an enriched environment induced better clearance of systemic infection in a model of sepsis without affecting the production of inflammatory markers. These effects are brought about by an increased recruitment of immune cells to the site of inflammation and a specific pattern of gene expression in whole blood that may be functionally associated with a swift return to an immunoprotective homeostatic state.

## Results

### Blood cellularity and weight changes in EE mice.

**Figure 1:**
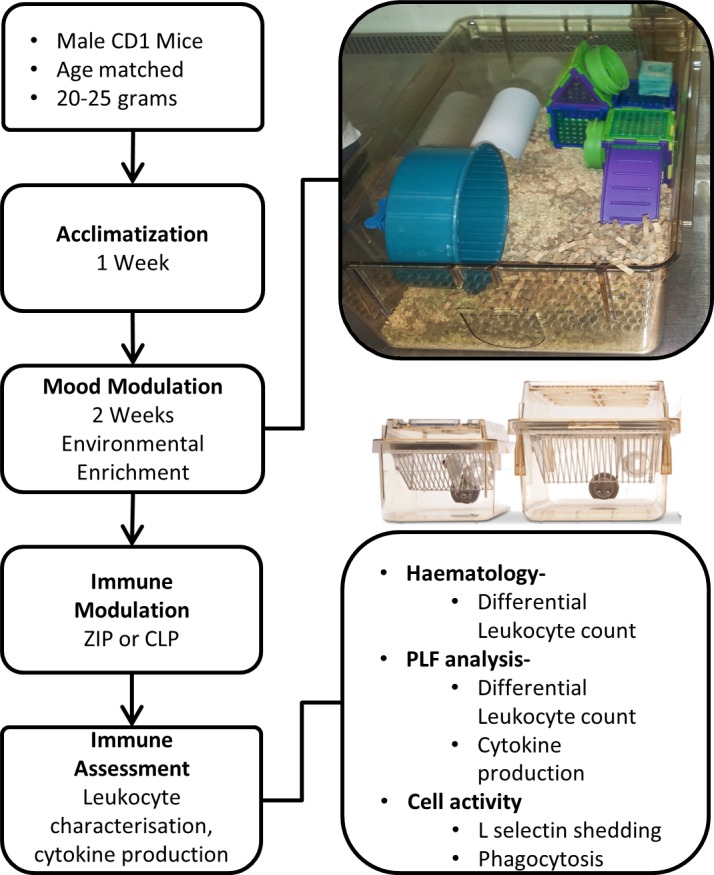
Experimental design, time line, and depiction of the enriched cage setup. Environmental enrichment conditions were kept consistent for all experiments. ZIP, zymosan-induced peritonitis; CLP, cecal ligation and puncture.

**Figure 2:**
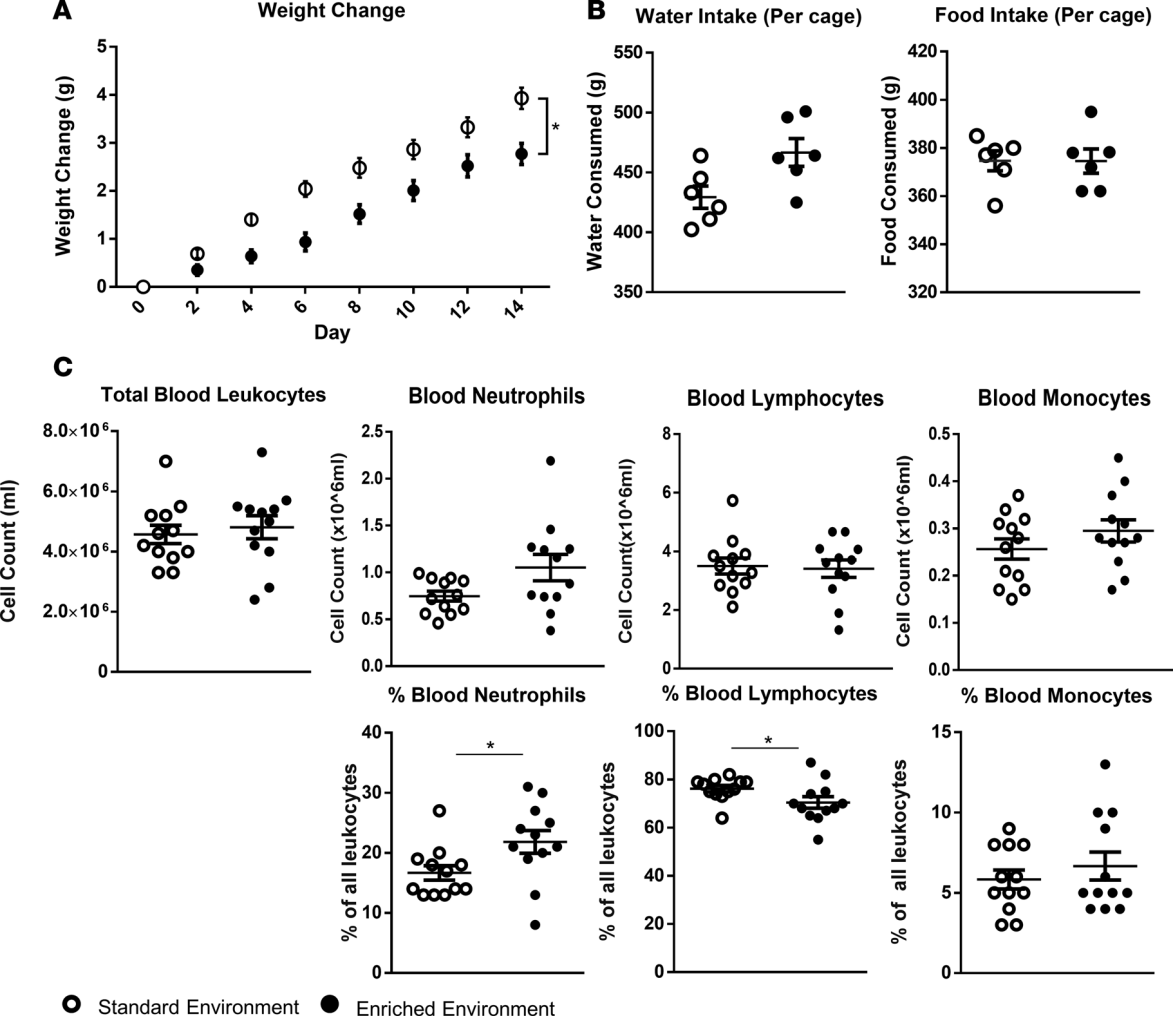
Altered metabolic and blood parameters in enriched mice. (**A**) (left to right) Environmentally enriched (EE) animals present a reduced rate of weight gain over a 2-week period compared with those housed in a standard lab environment (SE), while maintaining similar average food and water intake (**B**). (**C**) Total numbers of circulating leukocytes and relative percentages of leukocyte populations in the blood of EE and SE mice. Values are expressed as mean ± SEM of 12 mice per group and are representative of *n* = 3 independent experiments with similar results. *P < 0.05 by 2-tailed *t* test.

The overall experimental design is depicted schematically in Figure 1. Over the course of the 14-day enrichment period animals in the EE group consistently demonstrated a significantly lower weight gain compared with those from the SE (Figure 2A). Both groups maintained the same intake of food (average 27 g per group per day), while EE animals trended towards a slightly higher water intake (average 29 ml vs. 35 ml per day, respectively) (Figure 2B).

Peripheral analysis of blood samples taken at day 14 identified no difference in overall numbers of blood leukocytes or individual leukocyte populations between the 2 groups (Figure 2C). However, further assessment revealed a significant difference in the relative percentages of circulating cell types, with the blood of EE mice consistently showing a higher percentage of neutrophils and a correspondingly lower percentage of lymphocytes compared with the SE group (Figure 2D). No differences were found in relative levels of monocytes (Figure 2C).

### Zymosan-induced peritonitis in EE mice.

**Figure 3:**
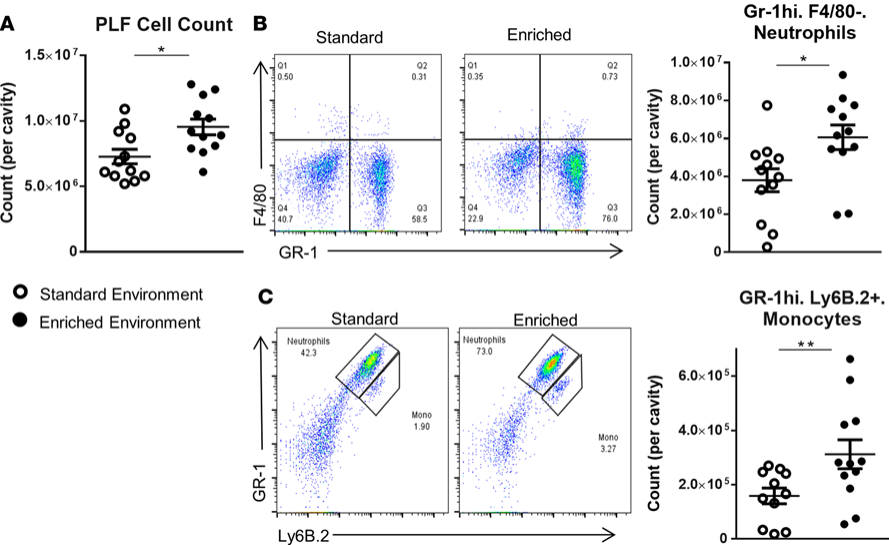
Increased peritoneal leukocytes in enriched mice following 6 hours of zymosan-induced peritonitis. (**A**) Peritoneal cell count of standard environment (SE) and environmental enrichment (EE) animals. PLF, peritoneal lavage fluid. (**B**) Representative FACS profiles of F4/80–expressing versus GR-1–expressing cells and comparative plots of total peritoneal populations of GR-1^hi^F4/80^–^ neutrophils in SE and EE mice. (**C**) Representative FACS profiles of Gr-1–expressing versus Ly6b.2-expressing cells and comparative plots of total peritoneal populations of GR-1^hi^Ly6b.2^+^ monocytes in SE and EE mice. Values are presented as individual data points with means (horizontal bars) ± SEM of 12 mice (with the exception of the enriched FACS plots in **B** and **C** where one sample was lost) and are representative of *n* = 3 experiments. *P < 0.05, **P < 0.005 by 2-tailed *t* test.

**Figure 4:**
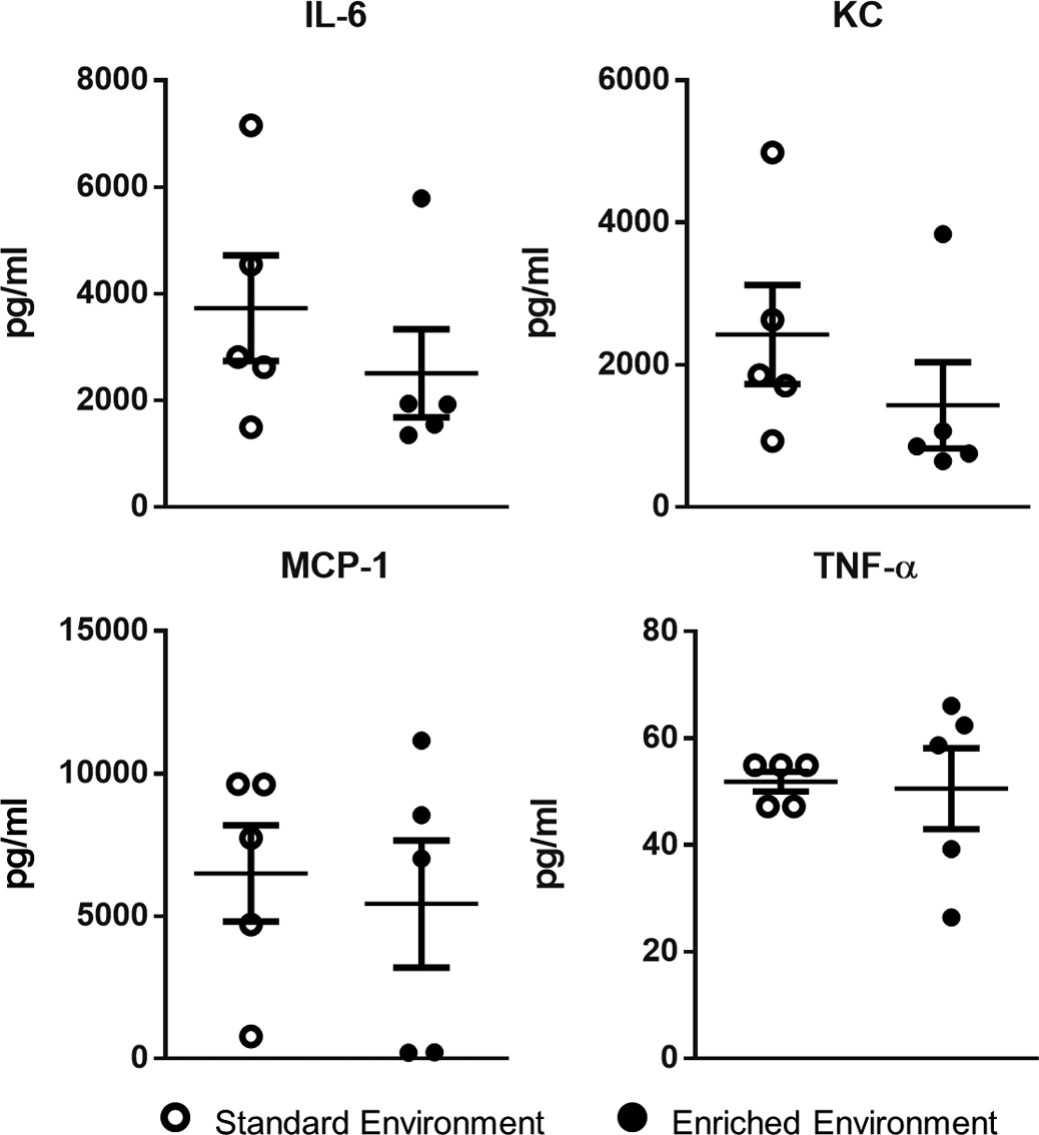
No difference in inflammatory cytokines in enriched mice following 6 hours of zymosan-induced peritonitis. Comparative expression of innate inflammatory cytokines IL-6, keratinocyte chemoattractant (KC), monocyte chemotactic protein 1 (MCP-1), and TNF-α in environmentally enriched and standard environment animals. Values are presented as individual data points with means (horizontal bars) ± SEM of 5 mice and are representative of *n* = 3 experiments.

We initially investigated differences in immune response between EE and SE animals using zymosan-induced peritonitis (ZIP), an experimental model of inflammation that allows the characterization of leukocyte egress into the peritoneal cavity. Six hours after zymosan injection, a significantly higher number of cells was found in the peritoneal lavage of EE animals (Figure 3A). FACS analysis identified the dominant cell population in both groups to be GR-1^hi^/F4/80^–^, ascribed as neutrophils (Figure 3B). Smaller populations of Gr-1^hi^/Ly6B.2^+^ cells, attributed as monocytes, were also identified (Figure 3C). Across both populations a significantly higher number of cells were present in the cavity of EE animals compared with those housed in the SE (scatter plots in Figure 3, B and C). Analysis of the collected peritoneal lavage fluid (PLF) identified heightened levels of the classical proinflammatory cytokines IL-6, keratinocyte chemoattractant (KC), monocyte chemotactic protein 1 (MCP-1), and TNF-α in the peritoneal cavity of mice from both groups; however, no significant difference was found in relative levels (Figure 4).

**Figure 5:**
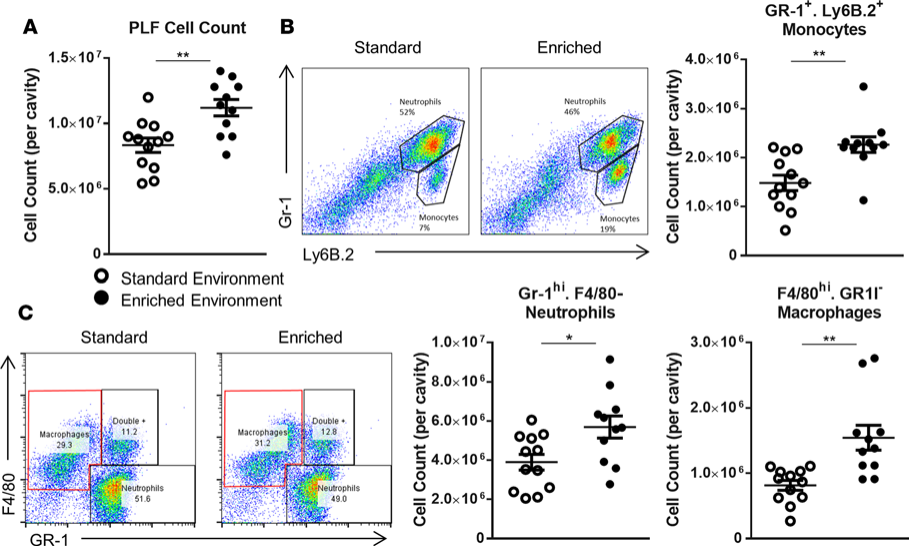
Increased peritoneal leukocytes in enriched mice following 24 hours of zymosan-induced peritonitis. (**A**) Peritoneal cell count of standard environment (SE) and enriched environment (EE) animals. PLF, peritoneal lavage fluid. (**B**) Representative FACS profiles of Gr-1–expressing versus Ly6b.2-expressing cells and comparative plots of total peritoneal populations of GR-1^hi^F4/80^–^ neutrophils in SE and EE mice. (**C**) Representative FACS profiles of F4/80–expressing versus GR-1–expressing cells and comparative plots of total peritoneal populations of GR-1^hi^Ly6b.2^+^ monocytes and F4/80^hi^Gr-1^–^ macrophages in SE and EE mice. Values are presented as individual data points with means (horizontal bars) ± SEM of 12 mice and are representative of *n* = 3 experiments. *P < 0.05, **P < 0.005 by 2-tailed *t* test.

**Figure 6:**
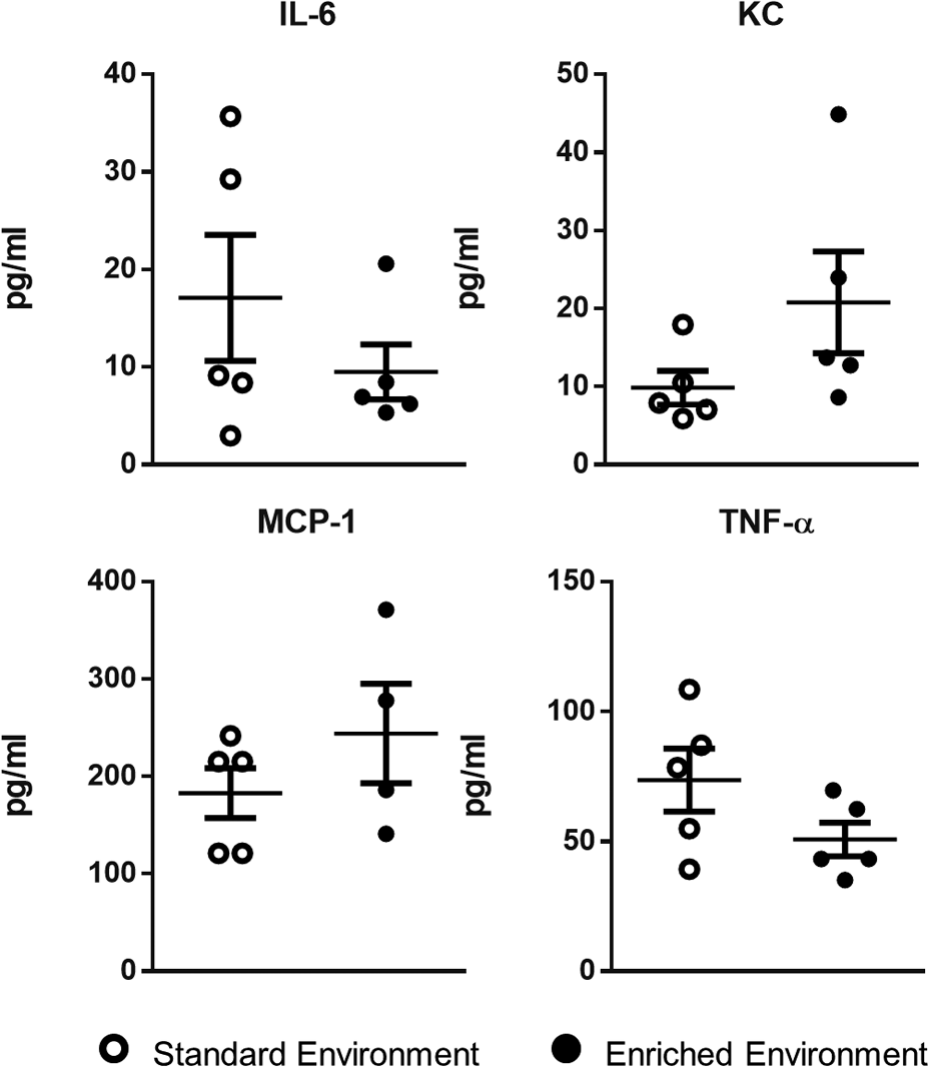
No difference in inflammatory cytokines in enriched mice following 24 hours of zymosan-induced peritonitis. Comparative expression of innate inflammatory cytokines IL-6, keratinocyte chemoattractant (KC), monocyte chemotactic protein 1 (MCP-1), and TNF-α in standard environment and environmentally enriched animals. Values are presented as individual data points with means (horizontal bars) ± SEM of 5 mice and are representative of *n* = 3 experiments.

At 24 hours after zymosan injection, EE animals still possessed a significantly higher number of peritoneal cells compared with those from the SE (Figure 5A). As with the 6-hour time point, FACS analysis identified higher numbers of GR-1^hi^/F4/80^–^ neutrophils and Gr-1^hi^/Ly6B.2^+^ monocytes in the EE group (Figure 5, B and C, respectively). An additional F4/80^hi^/Gr-1^–^ population, identified as monocyte-derived macrophages, was also found in both groups. Again, numbers of this population were significantly higher in EE animals (Figure 5C). Consistent with previous studies (20), analysis of the extracted PLF determined that proinflammatory cytokines levels had fallen greatly in both groups, with no difference in relative levels of expression (Figure 6).

### Effects of cecal ligation and puncture on EE mice.

**Figure 7:**
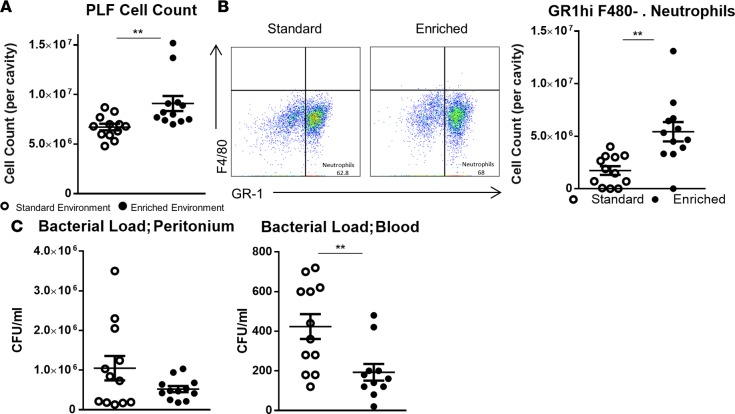
Enriched mice exhibit improved bacterial clearance in a experimental model of sepsis. (**A**) Peritoneal cell count of standard environment (SE) and environmentally enriched (EE) animals. PLF, peritoneal lavage fluid. (**B**) Representative FACS profiles of F4/80–expressing versus GR-1–expressing cells and comparative plots of total peritoneal populations of GR-1^hi^F4/80^–^ neutrophils in SE and EE mice. (**C**) Respective bacterial colony counts in peritoneal lavage fluid and blood of SE and EE animals values represented as CFU/ml. Values are presented as individual data points with means (horizontal bars) ± SEM of 12 mice and are representative of *n* = 2 experiments. **P < 0.005 by 2-tailed *t* test.

To assess the scale of the EE effect in a further model of peritoneal disease, we subjected EE and SE mice to cecal ligation and puncture (CLP) to produce sepsis. Twelve hours following the CLP protocol, a count of the extracted PLF presented a significantly higher number of cells in the peritoneum of EE mice compared with those from the SE (Figure 7A). FACS analysis attributed this increase in cell numbers to GR-1^hi^/F4/80^–^ neutrophils (Figure 7B).

**Figure 8:**
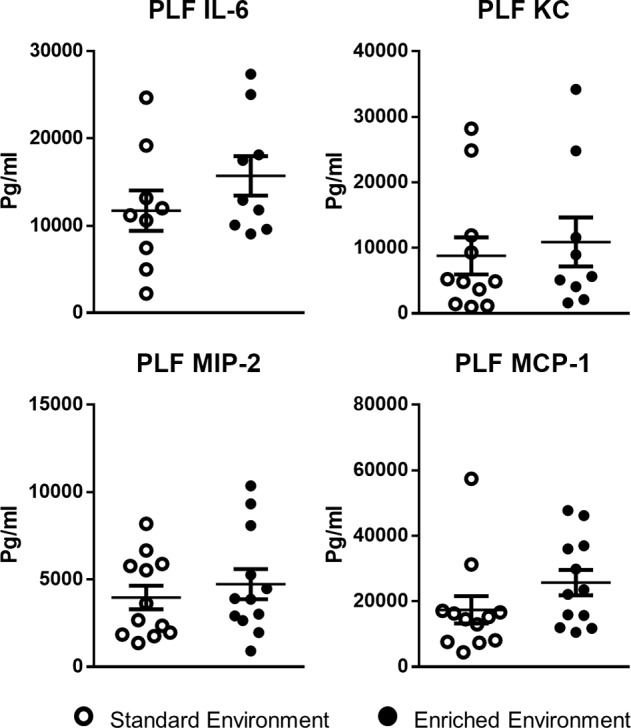
No difference in circulating inflammatory cytokines in enriched mice following 12 hours of sepsis induced by cecal ligation and puncture. Comparative expression of innate inflammatory cytokines IL-6, keratinocyte chemoattractant (KC), macrophage inflammatory protein 2 (MIP-2), and monocyte chemotactic protein 1 (MCP-1) in standard environment and environmentally enriched animals. Values are presented as individual data points with means (horizontal bars) ± SEM of 12 mice and are representative of *n* = 2 experiments. PLF, peritoneal lavage fluid.

Bacterial colony counts of the peritoneal fluids showed a trend (nonsignificant) for a lower overall bacterial count in the peritoneal cavity of EE animals (Figure 7B). Conversely, colony counts in blood were significantly lower in EE compared with SE mice, suggesting EE animals have a reduced systemic bacterial load compared with mice from the SE. As with the peritonitis model, analysis of extracted cell-free peritoneal exudates (Figure 8) and serum (data not shown) identified heightened levels of proinflammatory cytokines in both groups; however, no significant difference was found in relative levels.

### EE animals present heightened leukocyte activity ex vivo.

**Figure 9:**
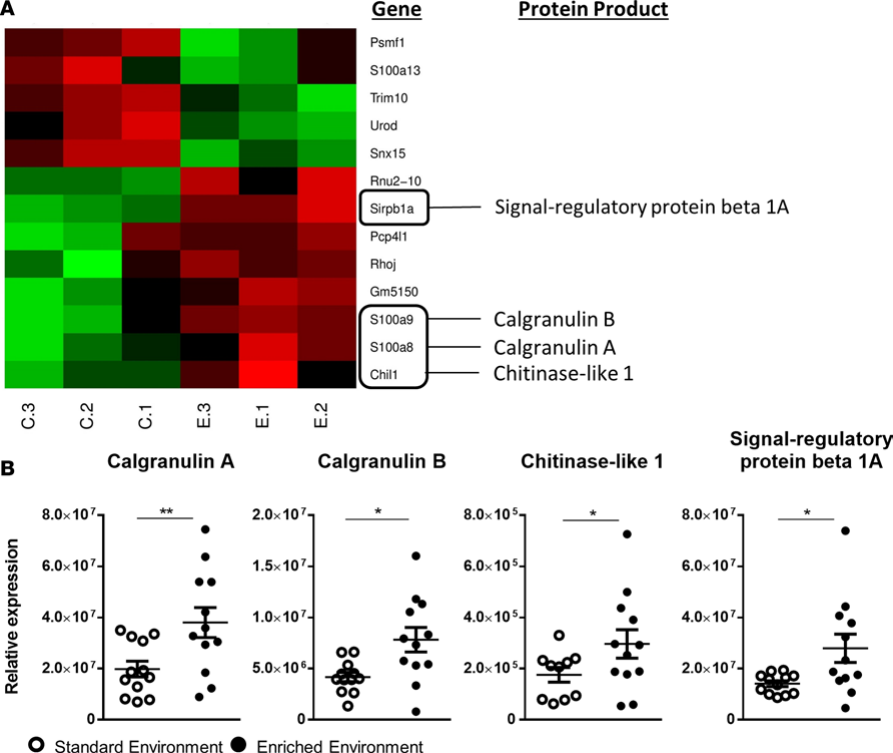
Enriched mice exhibit an altered immunoregulatory gene profile. (**A**) Heatmap analysis of microarray data of blood from standard environment and environmentally enriched mice. Hierarchical clustering and heatmap analysis of the filtered genes and the protein products of genes of interest. (**B**) Real-time PCR analysis of 4 genes of interest highlighted in the microarray. Values are presented as individual data points with means (horizontal bars) ± SEM of 12 mice. *P < 0.05, **P < 0.005 by 2-tailed *t* test.

To further investigate the altered immune response exhibited by EE animals, we subjected isolated leukocytes subsets from SE and EE mice to specific activity assays. Bio-Gel P-100–elicited macrophages isolated from the peritoneal cavity of EE and SE and animals had their capacity for phagocytosis assessed by culture with BODIPY-conjugated *E. coli* (dried). Macrophages isolated from EE mice were found to phagocytose a significantly higher quantity of bacteria over the course of the 1-hour incubation period than those from animals housed in the SE lab enclosure (Figure 9A). Additionally, the activation status of peripheral blood neutrophils was assessed by stimulation with platelet-activating factor (PAF) or TNF-α for 15 or 30 minutes. Following stimulation, cell populations were analyzed for the expression of activation markers CD11b and CD62L. A quantitative increase in the former and decrease of the latter (shedding) is considered indicative of a state of activation (21). No significant differences (calculated by 2-way ANOVA) were identified in the relative change of expression between both groups (Figure 9, B and C).

### EE mice present a significantly altered genetic profile.

**Figure 10:**
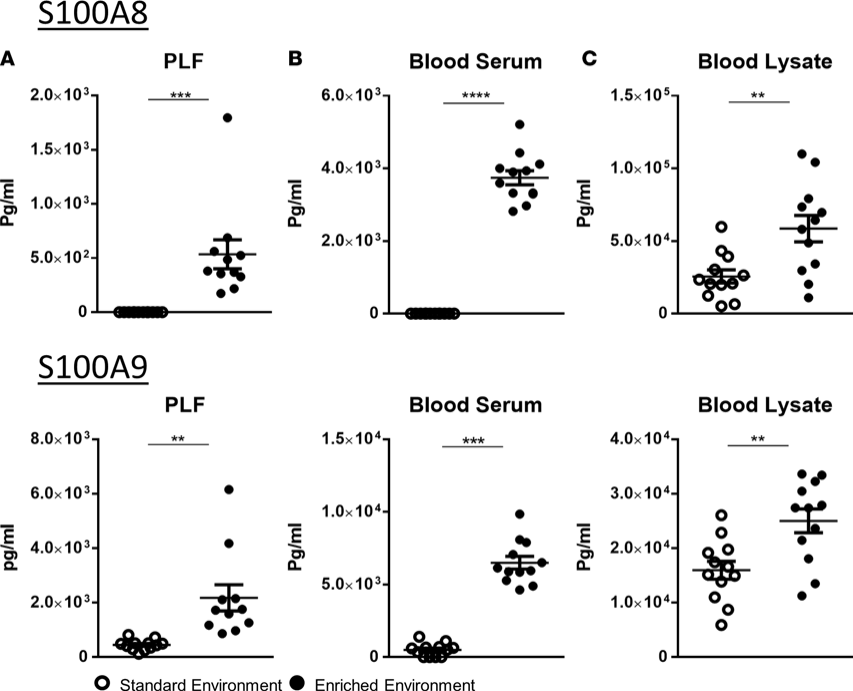
Enriched mice present heightened levels of circulating calgranulin. Comparative expression of the individual dimers of calprotectin (S100A8 and S100A9) in (**A**) peritoneal lavage fluid (PLF), (**B**) blood serum, and (**C**) blood pellet lysate. Samples were taken from environmentally enriched (EE) and standard environment (SE) mice subject to no additional immune modulation. Values are presented as individual data points with means (horizontal bars) ± SEM of 12 mice from SE and 11 for EE; *n* = 1 experiment. **P < 0.005, ***P < 0.001, ****P < 0.0005 by 2-tailed *t* test.

The functional association between EE and the efficiency of the inflammatory response brought us to identify underlying mechanisms responsible for this immune-modulating effect. To this end, we took a hypothesis-generating approach, comparing the genetic fingerprints of whole blood taken from EE and SE animals, subject to no inflammatory insult. Using fold change (FC) (>2 or <0.5) and non-adjusted *P* value less than 0.05, of the 34,760 probes present on the chip, 8 genes were identified as upregulated and 5 genes downregulated (Figure 10A). Of these genes, 4 of particular interest (s100a8, s100a9, chil1, and sirpb1a) were selected and their heightened expression confirmed via reverse transcription PCR (RT-PCR). A significantly higher level of gene expression (relative to the housekeeping gene GADPH) was identified in EE animals compared with those from the SE (Figure 10B).

**Figure 11:**
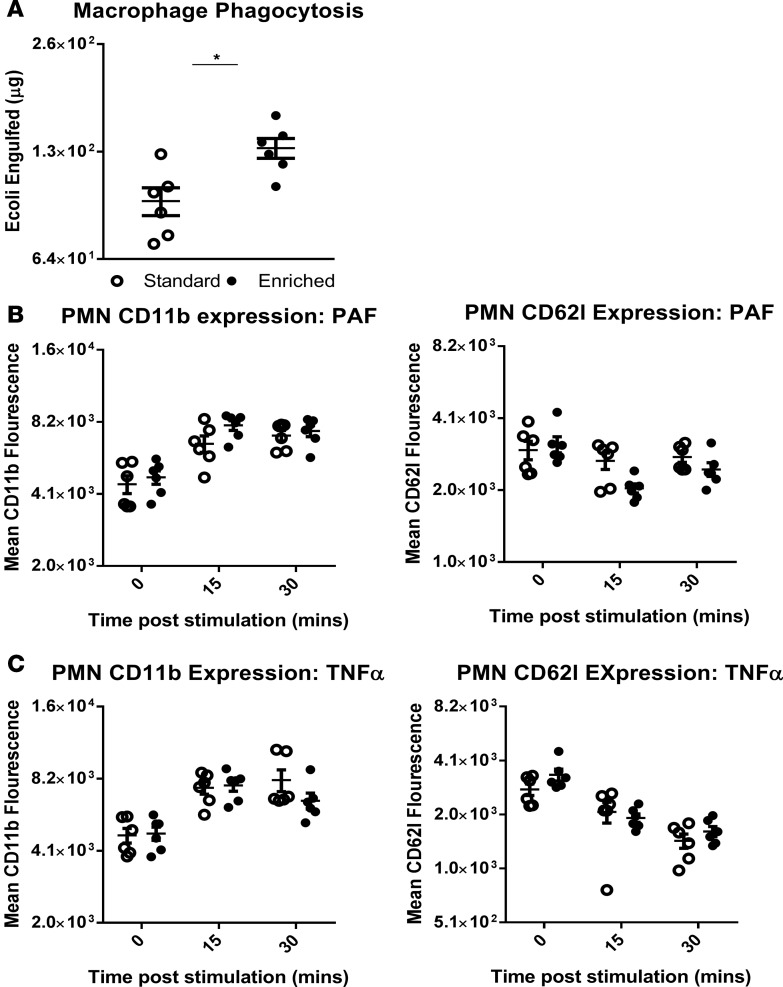
Differential ex vivo activity of leukocytes isolated from mice housed in a standard or enriched lab environment. (**A**) Comparative levels of BODIPY-linked *E. coli* phagocytosed by Bio-Gel P-100–generated macrophages in 1 hour were measured in mice housed in a standard (SE) or enriched environment (EE). EE macrophages were observed to engulf a significantly higher number of bacterial particles than SE. *P < 0.05 by 2-tailed *t* test. Representative of *n* = 3 experiments, 12 mice per experiment. (**B**) Comparative levels of CD11b and CD62L expression by activated polymorphonuclear leukocytes (PMNs) extracted from EE and SE animals were measured by flow cytometry. Following stimulation with either 10^−9^ M platelet activating factor (PAF) (**B**) or 50 ng/ml TNF-α (**C**) for 15 or 30 minutes, no differences in expression were detected. Values are presented as individual data points with means (horizontal bars) ± SEM of 6 mice and *n* = 2 experiments. Significance calculated (none found) by 2-way ANOVA.

To further confirm the gene signature of EE animals at the protein level, we measured the protein products S100A8 and S100A9 (calgranulin A and B, respectively) in the PFL (Figure 11A), blood plasma (Figure 11B), or total cell lysate (Figure 11C) of EE or control animals subject to no inflammatory insult. Consistent with the RT-PCR data, we observed significantly higher levels of these 2 soluble mediators in EE animals compared with SE.

## Discussion

This study sought to assess whether defined enhancements to an animal’s housing conditions could have a modulating effect on its immune status. We have used this experimental system to model the effects of external environmental factors on the inflammatory response and to investigate the possible underpinning cellular and molecular mechanisms behind these effects.

An enriched environment is known to have a beneficial effect on a variety of neurological parameters. Such research has revealed that EE is able to facilitate repair of neurological damage caused by chemical exposure (14) and physical trauma (15) and mitigate neurodegenerative disease (16). Alongside these neurological effects, a small number of studies (13, 17, 19, 22–24) have investigated the influence EE has on specific immune parameters, with some suggesting it may have an immune-enhancing effect in response to viral disease (17).

Our results show that EE invokes a significant and consistent alteration in the immune repertoire of male CD1 mice: increasing circulating innate immune cells, enhancing macrophage activity, and altering the host’s gene expression profile to improve bacterial clearance in a model of polymicrobial sepsis. These alterations are evident after only 2 weeks of enrichment, a time frame previously demonstrated to induce significant physiological effects in neurological research (25) and recently described in our previous study focusing on T cells (26).

We report that EE significantly boosts the cellular response in a model of ZIP. Intraperitoneal injection of zymosan induces a rapid accumulation of polymorphonuclear leukocytes into the peritoneal cavity. This immune response presents a consistent time course, made up of an initial influx of neutrophils 2–10 hours after injection followed by the later arrival of monocytes and monocyte-derived macrophages at 15 to 25 hours (20).

Differential analysis of the cell populations in EE revealed elevated numbers of neutrophils and monocytes compared with SE animals at 6 hours after challenge. Correspondingly, significantly higher numbers of peritoneal macrophages were found in EE animals 24 hours after zymosan injection. The initially higher influx of neutrophils into the EE animals mirrored the higher proportion we observed in circulation in basal conditions. In addition, the discovery of elevated numbers of macrophages and monocytes in the peritoneal cavity suggests an increased speed of egress to the site of inflammation. Lending support to this hypothesis, a previous study that subjected mice to enrichment for up to 18 weeks revealed that isolated leukocytes exhibit heightened proliferation and chemotaxis, indicating a common mode of effect for enrichment (13).

The results obtained from ZIP indicate that EE animals are able to mount a more effective inflammatory response, i.e., a response to the insult featured by increased clearance of the pathogenic stimuli (through increased recruitment of inflammatory cells) and a measured physiological production of inflammatory cytokines compared with SE animals. These results suggest that this heightened cellular response may not confer a corresponding increase in tissue damage in EE animals. To confirm this hypothesis, we applied a more clinically representative model of inflammation based on live bacterial infection — the CLP model of polymicrobial sepsis, which provides a much stronger insult able to elicit a marked local (peritoneal cavity) and systemic reaction (27). Our results show that EE animals are significantly more effective at clearing circulating bacteria than mice from the SE, and this was again associated with an increase in the number of peritoneal leukocytes. Combined with the ZIP data, increased numbers of peritoneal leukocytes are a common feature of the EE-evoked cellular response.

The results obtained from these 2 experimental systems, ZIP and CLP, led us to hypothesize that changes in external conditions are specifically translated into changes in the cellularity of the inflammatory response, ultimately leading to a better containment within the local tissue (in this case peritoneum) and hence a reduced transmission of microbes into the blood stream. It is interesting to note that previous studies have shown that diminished innate leukocyte activity and/or numbers are associated with a high mortality rate in sepsis (28, 29), consequent to immune paralysis and an overall failing of the innate response. Congruently, administration of the neutrophil attractants CXCL1 and CXCL2 into the peritoneal cavity after inducing polymicrobial sepsis enhances both neutrophil recruitment and clearance of bacteria, as well as improving host survival (30). Similarly, our recent study (25) on the influence of the enriched environment on T cell response ex vivo suggest that these cells might contribute to the beneficial effects of this experimental paradigm. Further studies will be needed to dissect the role of the innate and adaptive arms of the immune system in regulating the host immune response.

We were interested in further expounding the influence of EE on the immune system, and therefore carried out microarray analysis of RNA extracted from whole blood of EE and SE animals. Most of the genes identified by the array have specific metabolic or immunoregulatory function. Of these, 4 stood out for their role in inflammation: chil1 (chitinase-3-like protein 1, chi3l1), signal-regulatory protein β 1A (srbp1a), s100a8, and s100a9. Most interestingly, all of these genes are endowed with complex modulatory properties that both promote an efficient inflammatory response and contribute to host protection.

CHIL3L1 is serum protein predominately released by activated macrophages, neutrophils, endothelial cells, and astrocytes. Increased circulating levels of CHIL3L1 have been detected in patients suffering from rheumatoid arthritis (31), sepsis (32), and inflammatory bowel disease (33), suggesting that it plays a role in mediating inflammation. Supporting this hypothesis, CHI3L1 secretion is increased by leukocytes upon stimulation by inflammatory cytokines. Interestingly, CHI3L1 has also been shown to downregulate cellular responses to the same cytokines (31), indicating that CHI3L1 may be part of a feedback control mechanism regulating against an excessive inflammatory response. While the exact physiological role of CHIL3L1 is under ongoing research, studies have demonstrated its involvement in a range of immunological processes including apoptosis, inflammasome activation, M2 macrophage differentiation, TGF-β1 release, dendritic cell activation, and MAPK and Akt signaling (34).

Also upregulated was sirpb1a, a gene encoding the glycoprotein receptor SIRPB1A expressed on human monocytes and granulocytes (35). Stimulation of SIRPB1A on murine peritoneal macrophages has been shown upregulate phagocytosis (36), suggesting one possible mechanism for the upregulated macrophages we observed in this study. This activity has in turn been shown to play a protective role in the pathogenesis of Alzheimer’s disease by upregulating clearance of amyloid β aggregates through stimulation of phagocytosis by microglial cells (37). Further studies revealed that SIRPB1A binding upregulates neutrophil transendothelial migration (38), suggesting one possible explanation for the heightened leukocyte influx we observed in our CLP and ZIP models.

S100A8 and S100A9 together form the protein complex calprotectin that is highly abundant in myeloid cells, with neutrophils expressing the highest content (calculated to be ~30% of total cytosolic proteins). We elected to focus on this protein complex for protein confirmation in light of the commercial availability of reliable antibodies and because of its current interest to clinical research. Much of this research focuses on calprotectin’s role as a biomarker for inflammatory disease, most especially in intestinal inflammation (39) and more recently neonatal sepsis (40). Released by neutrophils, calprotectin exerts a direct antimicrobial action by sequestration of the essential bacterial nutrients manganese and zinc (41). The individual proteins have also been found to act as powerful regulators of the innate immune response by promoting neutrophil and macrophage accumulation as well as cytokine production (42, 43). Pharmacological administration of S100A8 to mice at the onset of a model of endotoxemia has been reported to significantly increase survival rate (44). Conversely, elevated calprotectin levels may contribute to early bacterial dissemination and liver injury in a model of *E. coli*–induced sepsis (45).

A further observation of this genetic analysis is that the spread of the data obtained for EE animals is notably greater than that obtained for SE. This variation may be attributed in part to the fact that CD1 mice are an outbred strain and that hence individual animals will possess an inherent variation in levels of gene expression. Another possibility is that EE itself leads to greater within-group variation.

This hypothesis has been addressed in several previous studies, with some authors suggesting the level of variation it causes to be negligible (46), while others posit that it leads to higher coefficients of variation in many readouts (47). One common reason cited for this variation is that EE leads to a greater level of intergroup fighting, probably due to increased territorial dispute and dominance displays (48, 49). Further studies would be needed to test the hypothesis that social dominance influenced gene expression variability in our experimental settings.

A great deal of research from our lab and many other groups has been invested in exploring mechanisms that favor or contribute to a more effective inflammatory response and a faster reestablishment of beneficial homeostasis. The results of this study provide a new outlook at the possible ways by which the immune response is able to reestablish a state of equilibrium, providing a mechanistic underscore to the impact of well-being reproduced herein with the EE exposure. With parallels to the established field of resolution of inflammation where lipids, short peptides, or gases have each been described as improving the outcome of an inflammatory response (50), we have revealed another strategy to achieve the same objective. According to this paradigm, the increased cellular response of the host might offer the opportunity to more efficiently respond to an immune insult while mitigating the damage of the inflammatory response. We also identified a specific network of signalling pathways associated with an immunoprotective phenotype that could be potentially used to design alternative therapeutic strategies to improve clinical outcome in the context of uncontrolled inflammation.

While EE appears to have a tangible effect on the immune response, deeper analysis is required to more fully understand if its effects could be of benefit in the context of disease and more specifically disease recovery. Most importantly for the progress of this field, efforts should be made to correlate and stratify clinical and animal studies with one another; only that way will a practicable benefit be derived from this research. EE has already been demonstrated to improve rodent wound healing in response to physical trauma (15), and comparable clinical studies have revealed that interventions designed to improve the mental well-being of patients led to a marked improvement in overall health (51) and reduced expression of genes associated with chronic systemic and cellular inflammation (52).

In this study, we aimed to further understand how the immune system as a whole specifically responds to changes in environmental conditions. The changes we observed at both molecular and cellular levels provide a link between enriched living conditions and an enriched immune response, i.e., an improved capability for the host to deal with inflammatory challenges.

These results are in line with a growing body of recent literature that has clearly demonstrated the need to investigate immune disorders and responses as complex multifactorial systems. These should include different factors such as living conditions and psychosocial status (53).

Perhaps the obvious question these findings raise is whether EE could serve as a viable treatment for human immune disease or as a prophylactic against microbial or inflammatory disease. The straight answer to this question is currently “no.” The results we have obtained cannot be directly translated from mice to humans. What can be learned from these data is that enriching the living conditions of experimental animals modulates the expression of a specific set of immune genes in a host, promoting an improved inflammatory response. Providing that we can verify these data in humans, one might use such genes to identify and stratify patients at risk of developing septicemia or other inflammatory pathologies. A far-reaching ambition for this study would be to pharmacologically modulate the immune genes we have identified to therapeutically treat high-risk patients.

Perhaps the most convincing conclusion to emerge from this study is that we cannot consider biological systems such as experimental animals or indeed human beings in all their diversity as fixed in their immune response. Both mice and humans respond proactively to changes in their living conditions. When we refer to proactively, we mean that both mice and humans change their metabolism, behavior, blood pressure, tissue regeneration, etc., according to changes in their living conditions. Such dynamic adjustments to an altering external and internal environment are often referred to as allostasis (54).

The ultimate purpose of animal experimentation is to emulate human physiological responses as accurately as possible. With the results of this study and others in mind, we suggest that a consideration of the environment in which we house such animals (55) and its parallels to human living conditions (53) should be an important part of experimental design.

## Methods

### EE protocol.

Upon receipt, male CD1 mice (Charles River) aged 6 weeks were placed in groups of 6 and given 1 week to acclimatize to the environment of the animal unit. During this time all groups were housed in a standard mouse enclosure consisting of 36 × 20 × 14 cm (523 cm^2^ horizontal area) cage (Allentown) with wood chip bedding and nesting material. Following this period, the groups were then rehoused in either the same standard enclosure setup (new cage, bedding, and nesting material) or in an enriched enclosure. EE enclosures were 50 × 38 × 21 cm (1,355 cm^2^ horizontal area, Allentown) and included 1 wheel, 1 nest house, 1 tunnel, ample nesting material, and wood chip bedding to a 5-cm depth.

All animal enclosures were cleaned out once per week. During cleaning mice were placed in a second enclosure along with all of their enrichment items as well as bedding. Roughly 20% of soiled bedding was left in the EE cage. After cleaning, all apparatus were replaced exactly as found. Prior to the onset of cleaning, mice were allowed a period of roughly 10 minutes to acclimatize to the presence of the researcher. No other researchers were allowed to handle the mice.

### ZIP.

Peritonitis was induced by i.p. administration of 0.5 mg of zymosan-A (Sigma-Aldrich) (16 mg/kg) diluted in sterile PBS. Six or 24 hours after injection mice were deeply anesthetized by isoflurane inhalation and blood was collected by cardiac puncture (anticoagulant: 3% solution of sodium citrate; approximately 1:10 ratio with blood). Immediately following blood extraction, animals were sacrificed by CO_2_ asphyxiation and a peritoneal lavage performed with 2 ml of cold PBS (4°C) containing 3 mM EDTA. PLF was used to measure cytokine levels using a Bio-Plex mouse cytokine assay (Bio-Rad) according to the manufacturer’s directions. Measurements were carried out using a Luminex Bio-Plex 200 system (Bio-Rad) and then analyzed with Bio-Plex Manager 6.1 software (Bio-Rad).

### Blood cellular and cytokine profiling.

Isolated blood was aliquoted and subject to hematological analysis using a ProCyte Dx Hematology Analyzer (IDEXX Laboratories). Plasma was obtained from a second blood aliquot set by centrifugation at 10,000 *g* for 3 minutes at 4°C. Concentrations of IL-6, MCP-1, TNF-α, macrophage inflammatory protein 2 (MIP-2), IFN-γ, S100A8, and S100A9 were determined using the Bio-Plex mouse cytokine assay as reported above.

### Flow cytometric leukocyte profiling.

Extracted PLF was centrifuged (377 *g*, 5 minutes, 4°C) and the pelleted leukocytes were resuspended in 1 ml of PBS. Following quantification by hemocytometer, leukocytes were washed and then stained in 100 μl of FACS buffer (PBS containing 5% FCS and 0.02% NaN_3_) containing CD16/CD32 FcγIIR–blocking antibody (clone 93; eBioscience) for 30 minutes at 4°C. Thereafter, cells were stained with the following FITC- or PE-conjugated antibodies (eBioscience): GR-1 (clone RB6-8C5), F4/80 (clone BM8), and Ly6B.2 (clone 7/4). Cells were labeled with the appropriate concentration of conjugated antibodies for 40 minutes at 4°C and then washed and analyzed. In all experiments, stained cells were acquired with an LSRFortessa flow cytometer (Becton Dickinson) and post-hoc analysis was carried out using FlowJo 7.0 software (Tree Star).

### Experimental sepsis by CLP.

Mice were deeply anesthetized by i.p. injection of a 1:1 solution of ketamine (75 mg/kg; Vetoquinol) and xylazine (15 mg/kg; Bayer). A midline laparotomy was performed to expose the cecum and adjoining intestine. The cecum was then ligated using 4.0 silk sutures (Prolene, Ethicon) just below the ileo-cecal valve and then punctured twice with a 15-gauge needle. Gentle pressure was applied to the cecum in order to extrude a small quantity of fecal matter from the sites of puncture. The cecum was then returned to the peritoneal cavity and the peritoneum and skin sutured closed. Mice were administered resuscitation fluid and allowed to recover from anaesthesia upon a heat pad and then returned to their cage. Animals were euthanized 12 hours later by terminal anaesthesia and subject to cardiac puncture, peritoneal lavage, and cellular analysis as described above. Isolated blood and PLF was streaked across nutrient broth (NB) agar plates (10-cm diameter) at varying dilutions from 10^–1^ to 10^–4^. The plates were incubated overnight, following which a count of the bacterial colonies growing on each plate was carried out. Colony-forming units per ml (CFU/ml) was calculated by multiplying the number of colonies counted by their dilution factor and dividing this figure by the volume of the culture plate. PFL cytokine levels were determined as detailed above.

All surgeries were conducted using aseptic techniques which meet at least the standards set out in the Home Office Minimum Standards for Aseptic Surgery. Consequently, postoperative infections were not expected. Any animal showing swelling, redness, reluctance to move, vocalization when handled, or posture suggestive of abdominal pain were sacrificed or referred to the named veterinary surgeon (NVS). Systemic analgesic was avoided because it might have interfered with the inflammatory/immune reaction (for instance, both innate and adaptive immune cells express receptors for opioids that are known to have a modulatory effect on these cells). Treatment was instituted on the advice of the NVS. The animals were euthanized by a Schedule 1 method if no improvement was seen in the first 12 hours of treatment.

### Microarray analysis.

Total RNA was extracted from blood of enriched (*n* = 3), and control (*n* = 3) mice using an RNeasy Protect Animal Blood System (Qiagen). Total RNA was hybridized to Affymetrix Mouse Gene 1.0 ST array chips at UCL Genomics with standard Affymetrix protocols, using a GeneChip Fluidics Station 450, and scanned using the Affymetrix GeneChip Scanner. Data were normalized by robust multiarray average (RMA) of the Bioconductor package, *affy*. Relevant genes were filtered by excluding those without an Entrez ID and those with low expression levels less than 100 by non-logged value. T-statistics were applied across the data set using the Bioconductor package Limma, and differentially expressed genes were identified by *P* < 0.05 (non-adjusted *P* value) and fold change greater than 2. Microarray data were approved and uploaded to the NCBI’s Gene Expression Omnibus database (GEO GSE94279).

### Real-time PCR.

Total RNA was extracted from blood (*n* = 12 for each mouse group) using the RNeasy Protect Animal Blood System according to the manufacturer’s protocol. Extracted RNA was reverse transcribed using 2 μg oligo(dT)15 primer, 10 U AMV reverse transcriptase, 40 U RNase inhibitor (all from Promega), and 1.25 mM each dNTP (Bioline) for 45 minutes at 42°C. The real-time PCR was carried out by using power SYBR GREEN master mix (Thermo Fisher Scientific) and QuantiTect primers (Qiagen). Cycling conditions were set according to the manufacturer’s instructions. The sequence-specific fluorescent signal was detected by a 7900HT Fast Real-Time PCR System (Applied Biosystems). Cycle threshold (Ct) values for each gene were normalized relative to glyceraldehyde 3-phosphate dehydrogenase (GAPDH) and then used to calculate expression levels. We used the comparative Ct method to measure the gene transcription in samples. Results are expressed as relative units based on calculation of 2−ΔΔCt, which gives the relative amount of a particular gene normalized to an endogenous control (GAPDH).

### Neutrophil activation.

Activation assays and analysis were carried out using previously published protocols (21). Briefly, blood samples were separated into 5 aliquots and treated respectively with TNF-α (50 ng/ml), PAF (10^−9^ M) for 15 or 30 minutes at 37°C, or left unstimulated. Samples were washed and stained for the presence of GR-1 APC (clone RB6-8C5), CDllb FITC (clone M1/M70) and PE-labeled L-selectin (CD62L) (clone Mel-14) on ice for 45 minutes (all antibodies were purchased from eBioscience). Each sample was lysed and fixed using an Immuno-Lyse reagent kit (Beckman Coulter). Neutrophils were gated according to forward/side scatter characteristics and positive expression of Gr-1 and CD11b. CD11b expression was recorded as units of fluorescence where the median fluorescence intensity for 10,000 cells was measured in the FL1 green channel (548 nm). In the case of CD11b antibody, the red FL2 channel was used (590 nm). Samples were analyzed by the BD LSRFortessa II with post-hoc analysis carried out using FlowJo 7.0 software.

### Phagocyte function.

The following procedure was based on previously reported methods (56). Animals were injected i.p. with 0.5 ml of a 4% solution of Bio-Gel P-100 (Bio-Rad) and left undisturbed for 4 days. Animals were then sacrificed and subject to peritoneal lavage with 10 ml of PBS. Collected cells were washed twice and then resuspended in complete RPMI-1640 media (Sigma-Aldrich). Isolated cells were confirmed as macrophages via microscopic analysis and then counted and seeded at 0.2 × 10^6^ cells per well in 200 μl of warm complete RMPI on a 96-well, black-walled, clear-bottom plate (Grenier Bio One) and allowed to adhere for 2 hours at 37˚C (5% CO_2_). Macrophages were then stimulated with BODIPY 576/589–conjugated (Invitrogen), lyophilized *E. coli* (Sigma-Aldrich) added at a final concentration of 1 mg/ml to each well. After 1 hour cell activity was halted and any non-phagocytosed *E. coli* were removed by washing 3 times with cold PBS. Fluorescence was read at 570 and 590 nm using a Multiskan FC Microplate Reader (Thermo Fisher Scientific).

### Statistics.

According to the nature of the data obtained, a *t* test (2-tailed), or ANOVA (1 or 2-way) was performed. Time-course observations were analyzed with multiple *t* tests and the Holm-Sidak post-hoc test. Behavioral data were analyzed via nonparametric analysis using the Mann-Whitney *U* test. All statistical analysis was performed using GraphPad PRISM software v6.0, with the exception of microarray analysis which was carried out as stated in 2.13.3 using the software package LIMMA (Bioconductor). Data were analyzed for normality using the D’Agostino-Pearson omnibus normality test.

### Study approval.

Adult male CD1 mice (5 weeks old) obtained from Charles River were used for all experiments. Animals were kept under standard conditions in individually ventilated enclosures, with food and water provided ad libitum and a 12-hour light/dark cycle. All animals were allowed a 7-day acclimatization period before any experimental procedure was performed upon them. All experiments were approved and performed under the guidelines of the Ethical Committee for the Use of Animals, Bart’s and The London School of Medicine and Home Office Regulations (57) (PPL 80/8714).

## Author contributions

SB designed research studies, conducted experiments, acquired and analyzed data, and wrote the manuscript. BG and TG provided technical assistance throughout the CLP studies. MO analyzed the acquired microarray data. MP provided advice on experimental design and editing of the manuscript. FD designed the research studies and assisted with conducting experiments.
